# Supplementary material for: The causal role of circulating inflammatory markers in osteoporosis: a bidirectional Mendelian randomized study
Source: Front Immunol. 2024 Jul 18;15:1412298. doi: 10.3389/fimmu.2024.1412298 (PMC11291241; doi:10.3389/fimmu.2024.1412298)
Supplement: Supplementary file 1 [file DataSheet_1.zip › supplementary material/OSTEOPOROSIS-CIM/GCST90274787.tsv.gz_leave_one_out.pdf]

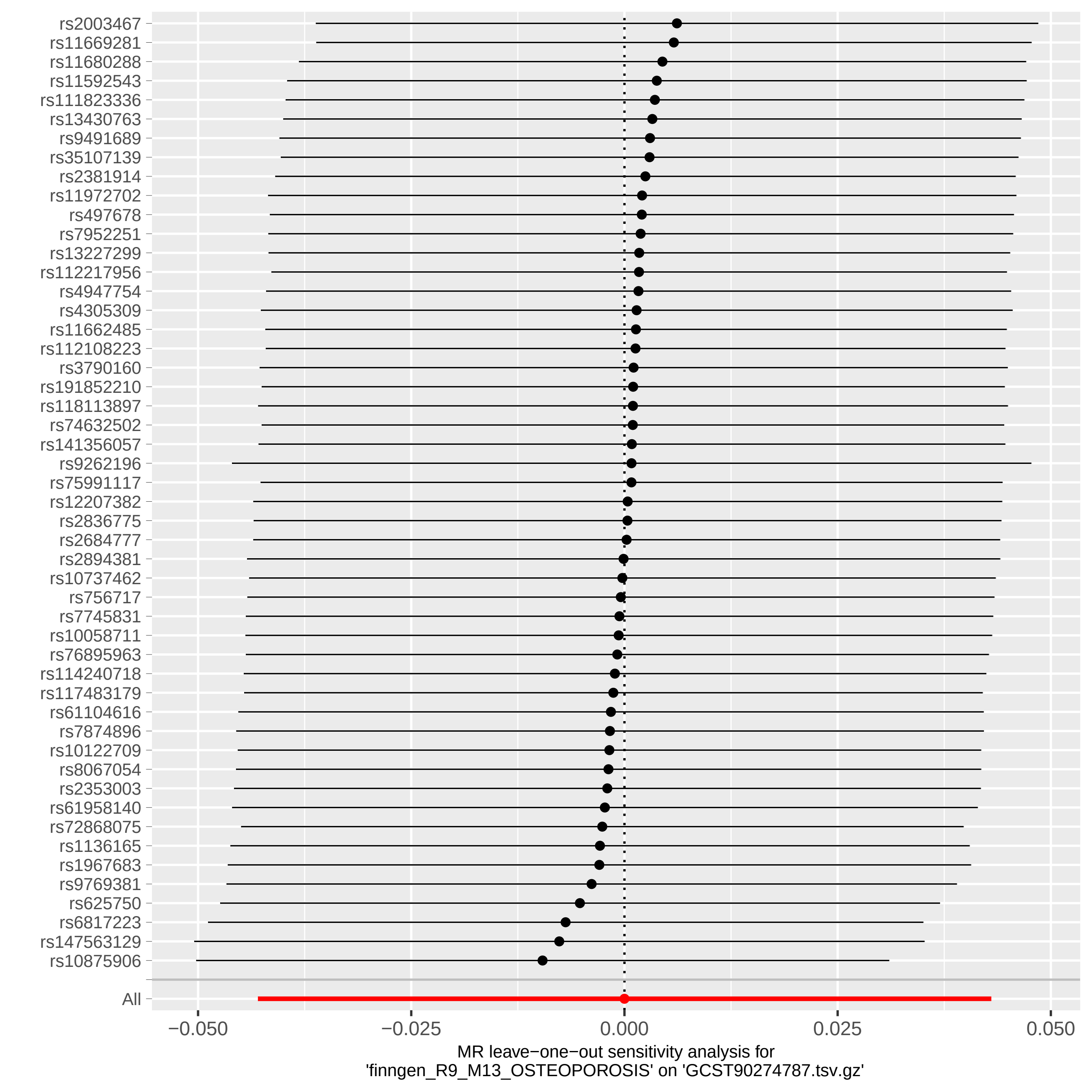
rs2003467
rs11669281
rs11680288
rs11592543
rs111823336
rs13430763
rs9491689
rs35107139
rs2381914
rs11972702
rs497678
rs7952251
rs13227299
rs112217956
rs4947754
rs4305309
rs11662485
rs112108223
rs3790160
rs191852210
rs118113897
rs74632502
rs141356057
rs9262196
rs75991117
rs12207382
rs2836775
rs2684777
rs2894381
rs10737462
rs756717
rs7745831
rs10058711
rs76895963
rs114240718
rs117483179
rs61104616
rs7874896
rs10122709
rs8067054
rs2353003
rs61958140
rs72868075
rs1136165
rs1967683
rs9769381
rs625750
rs6817223
rs147563129
rs10875906
All
−0.050
−0.025
0.000
0.025
0.050
MR leave−one−out sensitivity analysis for
'finngen_R9_M13_OSTEOPOROSIS' on 'GCST90274787.tsv.gz'
